# Hyaluronan synthase 2, a target of miR-200c, promotes carbon tetrachloride-induced acute and chronic liver inflammation via regulation of CCL3 and CCL4

**DOI:** 10.1038/s12276-022-00781-5

**Published:** 2022-06-03

**Authors:** Sun Myoung Kim, Ga Yeon Song, Aeri Shim, Jee Hyung Lee, Cheol Bin Eom, Cheng Liu, Yoon Mee Yang, Ekihiro Seki

**Affiliations:** 1 Department of Pharmacy, Kangwon National University, Chuncheon, 24341 South Korea; 2 KNU Researcher Training Program for Developing Anti-Viral Innovative Drugs, Kangwon National University, Chuncheon, 24341 South Korea; 3 Department of Infectious Disease, Putuo Hospital, Shanghai University of Traditional Chinese Medicine, 200062 Shanghai, China; 4 Karsh Division of Gastroenterology and Hepatology, Department of Medicine, Cedars-Sinai Medical Center, Los Angeles, CA 90048 USA

**Keywords:** Liver fibrosis, Chronic inflammation

## Abstract

Liver fibrosis occurs during wound healing after repeated liver injury and is characterized by extensive extracellular matrix deposition. We previously identified hyaluronan synthase 2 (HAS2) as a driver of liver fibrosis and hepatic stellate cell (HSC) activation. Developing strategies to suppress HSC activation is key to alleviating liver fibrosis, and HAS2 is an attractive candidate for intervention. To gain insight into the molecular function of HAS2, we investigated its posttranscriptional regulation. We found that miR-200c directly targets the 3’ untranslated regions of *HAS2*. Moreover, miR-200c and HAS2 were inversely expressed in fibrotic human and mouse livers. After establishing the direct interaction between miR-200c and HAS2, we investigated the functional outcome of regulating HAS2 expression in three murine models: CCl_4_-induced acute liver injury, CCl_4_-induced chronic liver fibrosis, and bile duct ligation-induced liver fibrosis. Hepatic *Has2* expression was induced by acute and chronic CCl_4_ treatment. In contrast, miR-200c expression was decreased after CCl_4_ treatment. HSC-specific *Has2* deletion reduced the expression of inflammatory markers and infiltration of macrophages in the models. Importantly, hyaluronidase-2 (HYAL2) but not HYAL1 was overexpressed in fibrotic human and murine livers. HYAL2 is an enzyme that can cleave the extracellular matrix component hyaluronan. We found that low-molecular-weight hyaluronan stimulated the expression of inflammatory genes. Treatment with the HA synthesis inhibitor 4-methylumbelliferone alleviated bile duct ligation-induced expression of these inflammatory markers. Collectively, our results suggest that HAS2 is negatively regulated by miR-200c and contributes to the development of acute liver injury and chronic liver inflammation via hyaluronan-mediated immune signaling.

## Liver fibrosis: Targeting polymer-producing enzyme could alleviate liver scarring

The molecular regulation of an enzyme involved in extracellular matrix production in liver tissues offers a promising therapeutic target for treating fibrosis. A team led by Ekihiro Seki from Cedars-Sinai Medical Center in Los Angeles, USA, and Yoon Mee Yang from Kangwon National University in Chuncheon, South Korea, found that a small regulatory RNA molecule called miR-200c represses the expression of hyaluronan synthase 2 (HAS2), an enzyme that stitches sugar molecules together to create a carbohydrate polymer found in the extracellular matrix that surrounds cells throughout the body, including the liver. The researchers found that miR-200c expression is reduced in fibrotic livers of humans and mice. This leads to elevated activity of HAS2. The increased production of hyaluronan polymers in turn promotes inflammatory signaling resulting in tissue fibrosis.

## Introduction

Liver fibrosis is characterized by excessive deposition of extracellular matrix components, such as collagen and hyaluronan (HA), as part of the wound healing response to repeated liver injury. Cirrhosis, an advanced stage of liver fibrosis, accounts for more than 1.3 million deaths per year worldwide^1^. The major causes of cirrhosis-related death include hepatitis B, hepatitis C, alcohol-related liver disease, and nonalcoholic steatohepatitis^1^. Although cirrhosis is the ninth leading cause of death in the United States, there are currently no FDA-approved drugs available for its treatment. Thus, there is an unmet need for antifibrotic drugs. Antifibrotic drug development has focused on the profibrogenic cytokine transforming growth factor beta (TGF-β), which activates extracellular matrix-producing hepatic stellate cells (HSCs). TGF-β also causes massive hepatocyte cell death that further contributes to liver fibrosis^2^. The development of antifibrotic therapies that target TGF-β isoforms or their receptors has been unsuccessful due to safety concerns, but approaches that target TGF-β downstream signaling still have potential^3^.

TGF-β regulates the biogenesis of several microRNAs (miRNAs)^4^, including the miR-200 family^5,6^. MiRNAs are small non-coding RNAs that are indispensable for gene regulation at the posttranscriptional level. The binding of miRNAs to the 3’ untranslated regions (3’UTRs) of their target genes results in mRNA degradation or translational repression^7^. Previous studies have shown that dysregulated miRNAs orchestrate pathological processes in liver diseases^8,9^. For example, miR-200c, a member of the miR-200 family, is downregulated in hepatocellular carcinoma and intrahepatic cholangiocarcinoma^10^. MiR-200c targets ZEB1, ETS1, and FLT1^11^, which are important regulators of epithelial-to-mesenchymal transition (EMT) and metastatic behavior. Thus, low miR-200c expression is associated with enhanced EMT and poor prognosis in cancer patients^11,12^. Reduced expression of miR-200c is also associated with the development of idiopathic pulmonary fibrosis^13^. In contrast, the expression of miR-200c suppresses EMT in alveolar epithelial cells, decreases the fibrogenic activity of TGF-β1 in lung fibroblasts, and attenuates pulmonary fibrosis^13^. Although miR-200c is negatively regulated by TGF-β and is implicated in both EMT and pulmonary fibrosis, its role in liver fibrosis is not fully understood.

Another downstream target of TGF-β is WT1^14^, which transcriptionally regulates hyaluronan synthase 2 (HAS2)^15^. HAS2 is responsible for the production of the extracellular matrix component HA and is highly expressed in activated HSCs^15^. High levels of HA, especially low-molecular-weight HA (LMW-HA, 100–300 kDa), are found in the serum and liver of patients and animals with liver fibrosis, and both HAS2 and HA promote HSC activation and fibrosis^15^. Thus, regulating the expression of HAS2 has potential antifibrotic value, and the study of its transcriptional and posttranscriptional regulation is warranted. HAS2 is also posttranscriptionally regulated by miRNAs, including miR-23^16^. However, the miRNA-mediated regulation of HAS2 in liver fibrosis has not been elucidated. Here, we report a predicted miR-200c binding site in the 3’UTR of HAS2.

The first step of HA synthesis is mediated by HAS2 and yields high-molecular-weight HA (HMW-HA), with a mass greater than 2000 kDa. Hyaluronidases, such as HYAL1, 2, 3, and 4, as well as TMEM2 and reactive oxygen species, degrade HMW-HA into LMW-HA^17,18^. LMW-HA stimulates fibrogenic gene expression as well as the production of several chemokines, including C-C motif chemokine ligand 3 (CCL3) and CCL4^19^. CCL3 (also called MIP-1α) and CCL4 (MIP-1β) belong to the macrophage inflammatory protein-1 (MIP-1) family and play important roles in immune cell recruitment. In the liver, CCL3 and CCL4 are produced by HSCs and immune cells, including macrophages and monocytes. CCL3 binds to the chemokine receptors CCR1, CCR4, and CCR5, whereas CCL4 specifically binds to CCR5. Importantly, the expression of CCL3 and CCL4, and their receptors CCR1 and CCR5 are elevated during liver fibrosis^20^, which promotes HSC migration and accelerates liver fibrosis^20,21^.

Here, we report a novel signaling axis involving miR-200c, HAS2, and inflammatory chemokines that contributes to liver fibrosis. Specifically, we found that miR-200c negatively regulates HAS2 expression and is downregulated in the fibrotic livers of patients and mice. Downregulation of miR-200c results in elevated HAS2 expression in HSCs, contributing to HA synthesis and fibrogenic gene expression. Moreover, we found that LMW-HA enhances the expression of CCL3 and CCL4 in HSCs and Kupffer cells, suggesting that HA promotes inflammation in the fibrotic liver environment. In summary, the present study demonstrates that HAS2 is posttranscriptionally regulated by miR-200c and that loss of this regulation in the liver results in inflammation and fibrosis via HA, CCL3, and CCL4 production.

## Materials and methods

### Materials

Recombinant human TGF-β was purchased from BioLegend (San Diego, CA, USA). Collagenase D, Collagenase P, polymyxin B, CCl_4_, 4-methylumbelliferone (4-MU), Fast Green FCF, and Direct Red 80 were supplied by Sigma–Aldrich (St. Louis, MO, USA). Nycodenz was obtained from Axis-Shiel (Oslo, Norway). Dulbecco’s modified Eagle’s medium (DMEM), RPMI 1640 medium, M199, and an antibiotic-antimycotic were purchased from Gibco (Grand Island, NY, USA). Rat tail Collagen I was purchased from Corning (Corning, NY, USA). The miR-200c mimic, miR-200c inhibitor, and corresponding controls were obtained from Bioneer (Daejeon, Korea). Fugene^®^ HD transfection reagent was purchased from Promega (Madison, WI, USA). The miRNA 3’UTR target expression clone for human NM_005328.2 (HAS2), miRNA target clone control vector for pEZX-MT06, and Luc-Pair Duo-Luciferase HS Assay Kit were supplied by GeneCopoeia (Rockville, MD, USA). Hyaluronic acid potassium salt derived from human umbilical cords was used as LMW-HA after confirming its size of ~200 kDa (ICN Biochemical, Inc., Aurora, OH, USA). HMW-HA (Healon) was obtained from Kabi Pharmacia Ophthalmics, Inc. (Monrovia, CA, USA). Biotin-labeled HA-binding protein [rhAggrecan aa20-675/His (NSO/7), biotin] was purchased from R&D Systems (Minneapolis, MN, USA). The anti-HYAL2 antibody and anti-CD31 antibody were obtained from Abcam (Cambridge, UK). The anti-Desmin antibody was purchased from Santa Cruz Biotechnology (Santa Cruz, CA, USA). Rat anti-F4/80 and mouse anti-α-SMA monoclonal antibodies were provided by eBioscience (San Diego, CA, USA) and Dako (Carpinteria, CA, USA), respectively. M.O.M., VECTASTAIN Elite ABC, and DAB Peroxidase Substrate kits were obtained from Vector Laboratories (Burlingame, CA, USA). The Alexa Fluor^®^ 594-conjugated donkey anti-rabbit IgG (H + L) secondary antibody, Alexa Fluor^®^ 488-conjugated donkey anti-rat IgG (H + L) secondary antibody, Alexa Fluor^®^ 488-conjugated donkey anti-goat IgG (H + L) cross-adsorbed secondary antibody, SlowFade^TM^ Diamond Antifade Mountant with DAPI, and Blocker^TM^ Casein in PBS were obtained from ThermoFisher Scientific (Carlsbad, CA, USA).

### Animals

All animal protocols were approved by the Institutional Animal Care and Use Committee (IACUC) of Cedars-Sinai Medical Center. Male C57BL/6 J mice were purchased from The Jackson Laboratory (Bar Harbor, ME, USA). After mice were acclimatized for one week, experiments were conducted in accordance with institutional guidelines. To generate HSC-specific *Has2* knockout (*Has2*^Δ*HSC*^) mice, *Has2*^*flox/flox*^ mice were crossed with *Lrat*-Cre transgenic mice, as previously described^15^. HSC-specific *Has2* deletion was confirmed. Floxed littermates without Cre expression were used as wild-type controls. To establish the model of CCl_4_-induced acute liver injury, C57BL/6 J or *Has2*^Δ*HSC*^ mice and their wild-type controls were injected intraperitoneally with one dose of corn oil or CCl_4_ (0.5 μl CCl_4_/g mouse body weight, 1:4 dilution with corn oil). To establish the model of CCl_4_-induced chronic liver fibrosis, CCl_4_ was administered twice a week for 6 or 8 weeks at the same dose. To establish the model of bile duct ligation (BDL)-induced cholestatic liver disease, mice were anesthetized with ketamine and xylazine. BDL was performed as previously described^15^. Sham-operated mice were similarly laparotomized, but double ligation of the common bile duct with surgical sutures was not performed. Liver tissues were harvested 5 days or 21 days after surgery. In another experiment, 4-MU treatment (225 mg/kg, P.O., twice a day) was initiated on the day of the BDL operation. Five days after BDL, mice were sacrificed. We also analyzed samples from the livers of wild-type and *Has2*^Δ*HSC*^ mice subjected to sham or BDL operation from a previous study^15^.

### Primary hepatic stellate cell, Kupffer cell, and hepatocyte isolation and treatment

HSCs and Kupffer cells were isolated in a two-step process that included pronase-collagenase perfusion of mouse livers and Nycodenz density gradient centrifugation, as previously described^15,22^. We used magnetic antibody cell sorting (MACS; Miltenyi Biotec, Bergisch-Gladbach, Germany) with CD11b MicroBeads (Miltenyi Biotec) to isolate Kupffer cells with high purity according to the manufacturer’s instructions. Isolation of HSCs or Kupffer cells with high viability (>95%) was achieved, as determined by trypan blue staining. HSCs were maintained in DMEM supplemented with 10% fetal bovine serum (FBS) and 1% antibiotic-antimycotic at 37 °C in a humidified atmosphere containing 5% CO_2_. On Day 3, HSCs were serum-starved overnight and were then treated with TGF-β (5 ng/ml) alone or with LMW-HA (100 or 200 μg/ml) or HMW-HA (600 μg/ml) in combination with 10 μg/ml polymyxin B for 12 h. Isolated Kupffer cells were seeded in RPMI 1640 medium containing 10% FBS and 1% antibiotic-antimycotic. A total of 2 × 10^5^ cells/well were plated in 12-well plates. After 4–6 h, the medium was changed to serum-free medium, and the cells were incubated overnight. Subsequently, Kupffer cells were treated with LMW-HA (100 or 200 μg/ml) or HMW-HA (600 μg/ml) in combination with 10 μg/ml polymyxin B for 4 h. Primary hepatocytes were isolated from mice by in situ liver perfusion with Collagenase D and Collagenase P. After enzymatic digestion, the cell suspension was filtered through a 70 μm nylon mesh and was then centrifuged at 50 × *g* for 1 min at 4 °C. Dead cells were removed by centrifugation at 150 × *g* for 7 min over a 15 ml cushion of a 33% Percoll solution. The viability of hepatocytes isolated from corn oil-treated mice was 95.1%, and the viability of hepatocytes isolated from CCl_4_-treated mice was 88.8%. Hepatocyte pellets were resuspended in M199 supplemented with 10% FBS and 1% antibiotic-antimycotic. A total of 5 × 10^5^ cells/well were plated on collagen-coated plates.

### Quantitative polymerase chain reaction (qPCR)

Total RNA was extracted using a NucleoSpin^®^ RNA kit (Macherey-Nagel, Düren, Germany) or TRIzol (Invitrogen, Carlsbad, CA, USA). DNase I-treated RNA was reverse-transcribed using a High-Capacity cDNA Reverse Transcription Kit (Applied Biosystems, Foster City, CA, USA) or a miScript RT Kit (Qiagen, Hilden, Germany) according to the manufacturer’s recommendations. The expression levels of the genes of interest were determined with iTaq™ Universal SYBR^®^ Green Supermix (Bio–Rad, Hercules, CA, USA) or a miScript SYBR Green PCR Kit (Qiagen). The sequences of the PCR primers used in this study are presented in Supplementary Table 1. PCR cycling was conducted on a CFX96 real-time PCR system (Bio–Rad).

### Transfection

LX-2 cells were transfected with the miRNA mimic (100 nM) or miR-Down Antagomir (100 nM) using FuGENE HD Reagent. Cells were harvested 72 h after transfection, and qPCR experiments were performed. For luciferase assays, cells were cotransfected with the *HAS2* 3’UTR luciferase vector (or with pEZX-MT06 as a miRNA target control vector) and the miR-200c mimic (or control mimic). After 48 h, luciferase activity was assessed using a Luc-Pair Duo-Luciferase HS Assay Kit (GeneCopoeia, Rockville, MD, USA) in accordance with the manufacturer’s instructions.

### Human liver samples

Liver samples from 65 patients with chronic HBV infection and clinically diagnosed liver fibrosis were collected between February 2013 and July 2016 in Putuo Hospital, Shanghai University of Traditional Chinese Medicine (Shanghai, China) in accordance with the ethical guidelines of the 1975 Declaration of Helsinki, as previously described^15^. The institutional review board approved the study protocol and written informed consent was obtained from each participating subject.

### Immunofluorescence staining

Frozen liver sections were sliced into 5 μm-thick sections. After fixation, the tissue sections were blocked with Blocker^TM^ Casein in PBS. The samples were incubated with an anti-HYAL2 antibody and a cell-type-specific antibody against F4/80, Desmin, or CD31 at 4 °C overnight. After washing, the samples were incubated first with an Alexa Fluor 488-conjugated secondary antibody for 1 h at room temperature, and then with an Alexa Fluor 594-conjugated donkey anti-rabbit IgG (H + L) secondary antibody for 1 h. After washing, the samples were mounted in SlowFade^TM^ Diamond Antifade Mountant with DAPI. Confocal images were acquired with an LSM880 supersensitive high-resolution confocal laser scanning microscope (Carl Zeiss) and analyzed with Zeiss Zen microscopy software.

### Histopathology

Mouse liver tissues were fixed with 10% buffered formalin for 24–48 h before embedding in paraffin. Paraffin-embedded tissue specimens were sectioned at 5 μm using a microtome. Hematoxylin and eosin (H&E) staining was performed following a basic protocol provided by Leica Biosystems. Briefly, samples were dewaxed and rehydrated. Then, the samples were sequentially immersed in hematoxylin, differentiation solution, bluing agent, and eosin. The samples were dehydrated in 95% and 100% ethanol and were then cleared in xylene solution. Finally, the samples were mounted with a coverslip.

For Sirius Red staining, Picrosirius Red solution was prepared by dissolving Fast Green FCF and Direct Red 80 in saturated picric acid solution. After deparaffinization and rehydration, Picrosirius Red solution was applied to liver sections and incubated for 1 h. The sections were rinsed with acetic acid solution two times and subsequently washed with absolute alcohol. The sections were dehydrated in two changes of absolute alcohol and were then mounted.

Immunochemistry for F4/80 and α-SMA was performed as previously described^15^. Briefly, a target retrieval solution (Dako) was applied to deparaffinized and rehydrated sections for heat-induced antigen/epitope retrieval. Endogenous peroxidase activity was blocked by submerging slides in 3% hydrogen peroxide for 30 min. Residual biotin, avidin, or streptavidin activity was quenched by sequential incubation with avidin solution and d-biotin solution. Slides were incubated with a rat anti-mouse F4/80 antibody or a mouse anti-human α-SMA antibody in antibody diluent. A M.O.M. kit was used according to the manufacturer’s instructions for α-SMA. After primary antibody incubation, a secondary antibody was applied. Streptavidin-horseradish peroxidase reagent was added, and staining was visualized by applying DAB solution. Hematoxylin was used as a counterstain. For HA staining, biotin-labeled HA-binding protein was applied. Images were acquired at ×100 or ×200 magnification. NIH ImageJ software (Bethesda, MD, USA) was used for quantification.

### Statistical analysis

SigmaPlot 10.0 (Systat Software, Point Richmond, CA, USA) or GraphPad Prism 8.01 software (GraphPad Software, Inc., La Jolla, CA, USA) was used for statistical analysis. Groups were compared with either Student’s *t-*test or one-way ANOVA followed by Tukey’s post-hoc test. *P*-values <0.05 were considered statistically significant. Data are presented as the mean ± SEM values.

## Results

### Identification of miRNAs associated with liver fibrosis

**Fig. 1:**
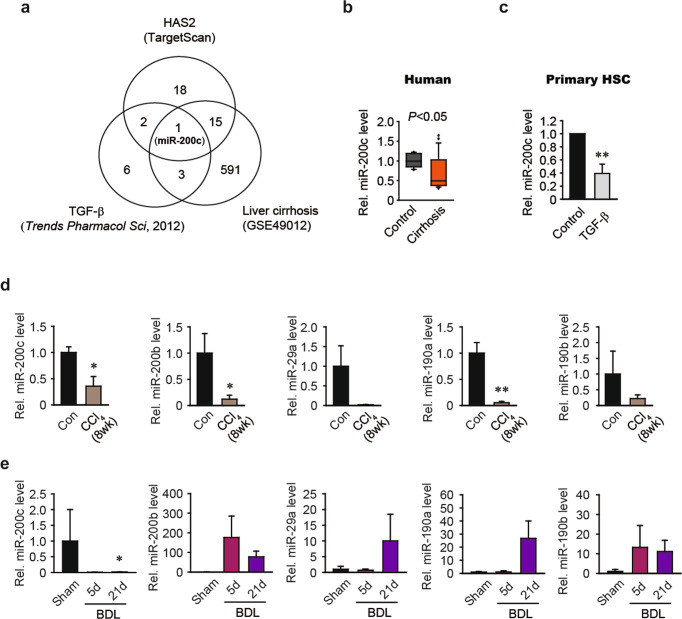
The expression of miR-200c is reduced in liver fibrosis. **a** Venn diagram showing the overlapping miRNAs. The number of miRNAs computationally predicted by TargetScan to target HAS2 is shown in the upper circle. The number of miRNAs downregulated by TGF-β administration^4^ is shown in the lower left circle. The number of miRNAs downregulated in liver tissues from individuals with cirrhosis (GSE49012) is shown in the lower right circle. MiR-200c is depicted in the three overlapping circles. **b** MiR-200c transcript levels in liver tissues from patients. We reanalyzed GEO dataset GSE49012 (Control, *n* = 12; Cirrhosis, *n* = 22). **c** Quantitative reverse transcription-polymerase chain reaction (qRT–PCR) analysis of miR-200c expression after TGF-β treatment in mouse primary HSCs. HSCs were treated with TGF-β for 12 h. **P < 0.01 versus the control group (*n* = 4). **d** Hepatic levels of miR-200c, miR-200b, miR-29a, miR-190a, and miR-190b. Mice were injected intraperitoneally with corn oil (Con) or carbon tetrachloride (CCl_4_) twice a week for 8 weeks. *P < 0.05, **P < 0.01 versus the Con group (*n* = 4–5 mice/group). **e** Quantification of hepatic miRNA levels. Mice underwent bile duct ligation (BDL) and were assessed after 5 or 21 days. *P < 0.05 versus the sham group (Sham, *n* = 2; BDL 5d, *n* = 4; BDL 21d, *n* = 8). The values shown are the means ± SEMs. Significance was assessed by two-tailed Student’*s t*-test (**b–d**) and one-way ANOVA with Tukey’s post-hoc test (**e**).

To identify the miRNA(s) responsible for the upregulation of HAS2 in liver fibrosis, we searched TargetScan 7 for putative miRNAs that target HAS2. Specifically, we searched for 8-mer or 7-mer m8 sites with exact matches at positions 2–8 that were broadly conserved among vertebrates. A search with these criteria returned 36 miRNA candidates (Fig. 1a). We previously found that TGF-β, a profibrogenic cytokine, induces HAS2 expression in HSCs^15^. Interestingly, TGF-β was reported to reduce the expression of three of our putative miRNAs: miR-200a, miR-200b, and miR-200c (Fig. 1a)^4^. As miRNAs negatively regulate gene expression, we were interested in those that were downregulated under fibrotic conditions. We further analyzed miRNA profiles in liver tissues collected from individuals with liver cirrhosis (*n* = 22) during liver transplantation and in normal liver tissues (*n* = 12) collected from patients who underwent liver resection surgery for metastatic/benign lesions that were included in a publicly available microarray dataset (GSE49012)^23^. Six hundred ten miRNAs were significantly downregulated (fold change, <0.7) in the liver cirrhosis samples compared to the normal liver samples. Of the putative miRNAs that target HAS2, only miR-200c was downregulated both by TGF-β treatment and in cirrhotic liver samples (Fig. 1a). MiR-200c expression was reduced in liver samples from patients with liver cirrhosis compared to normal liver tissues (Fig. 1b). Moreover, real-time PCR analysis verified that the miR-200c level was reduced in primary mouse HSCs treated with TGF-β when compared to the control cells (Fig. 1c). As miR-200c was the most likely regulator of HAS2 in the fibrotic liver, we focused our subsequent investigation on the signaling network that connects TGF-β, miRNA, HAS2, and liver fibrosis.

We began by examining miR-200c expression in murine models of liver fibrosis. We observed that hepatic miR-200c levels were significantly decreased in CCl_4_-treated mice compared to corn oil-treated mice (Fig. 1d). We further examined miR-200c expression levels in a murine model of BDL-induced liver fibrosis. Consistent with the findings in the CCl_4_-induced liver fibrosis model, miR-200c levels were markedly decreased in the livers of mice with BDL-induced liver fibrosis 21 days after BDL (Fig. 1e). We also assessed the expression of miR-200b, which has the same seed sequence as miR-200c and is also downregulated by TGF-β^4^. Although miR-200b expression was reduced in the CCl_4_-induced liver fibrosis model (Fig. 1d), its expression level was not altered in the BDL-induced liver fibrosis model (Fig. 1e). We observed similar trends in the expression of other HAS2-targeting miRNA candidates, including miR-29a, miR-190a, and miR-190b (Fig. 1d, e). Our results suggest that only miR-200c is downregulated in both human and mouse liver fibrosis.

### MiR-200c is a novel inhibitor of HAS2

**Fig. 2:**
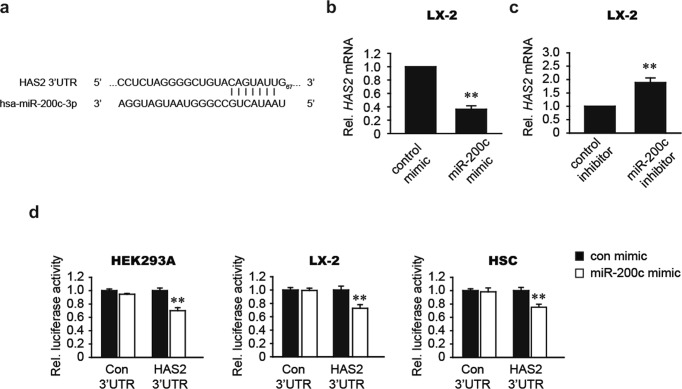
miR-200c directly targets HAS2. **a** Prediction of miR-200c binding to the 3’UTR of human *HAS2* mRNA. **b** The effect of miR-200c mimic treatment on the *HAS2* mRNA level. **c** The effect of miR-200c inhibitor treatment on the *HAS2* mRNA level. **d** The effect of miR-200c mimic treatment on pEZX-*HAS2*-3’UTR luciferase activity. Luciferase activity was assessed in HEK293A cells, LX-2 cells, and mouse HSCs transfected with the negative control mimic or miR-200c mimic in combination with pEZX-control or pEZX-*HAS2*-3’UTR. **P < 0.01 indicates a significant difference between the control mimic (or inhibitor) and miR-200c mimic (or inhibitor) (*n* = 3–6 per group). The data are presented as the mean ± SEM values. Significance was assessed by two-tailed Student’s *t-*test (**b, c**) and one-way ANOVA with Tukey’s post-hoc test (**d**).

TargetScan identified a putative miR-200c binding site in the 3’UTR of *HAS2* (Fig. 2a). To determine the effect of miR-200c on *HAS2* mRNA regulation, the human HSC LX-2 cell line was transfected with either the miR-200c mimic or scrambled control miRNA. Transfection with the miR-200c mimic markedly decreased the *HAS2* mRNA level (Fig. 2b). In contrast, transfection with the miR-200c inhibitor significantly increased the *HAS2* mRNA level (Fig. 2c). To clarify whether HAS2 is a direct target of miR-200c, we utilized a pEZX-*HAS2*-3’UTR luciferase construct (Fig. 2d). We evaluated luciferase activity in LX-2 cells, HEK293A cells, and mouse HSCs and found that the miR-200c mimic reduced the luciferase activity of the pEZX-*HAS2*-3’UTR in all three cell types. Transfection of miR-200c did not alter luciferase activity in pEZX-control-transfected cells (Con 3’UTR). Together, these results suggest that miR-200c directly targets HAS2.

### miR-200c suppresses fibrogenic and inflammatory gene expression

**Fig. 3:**
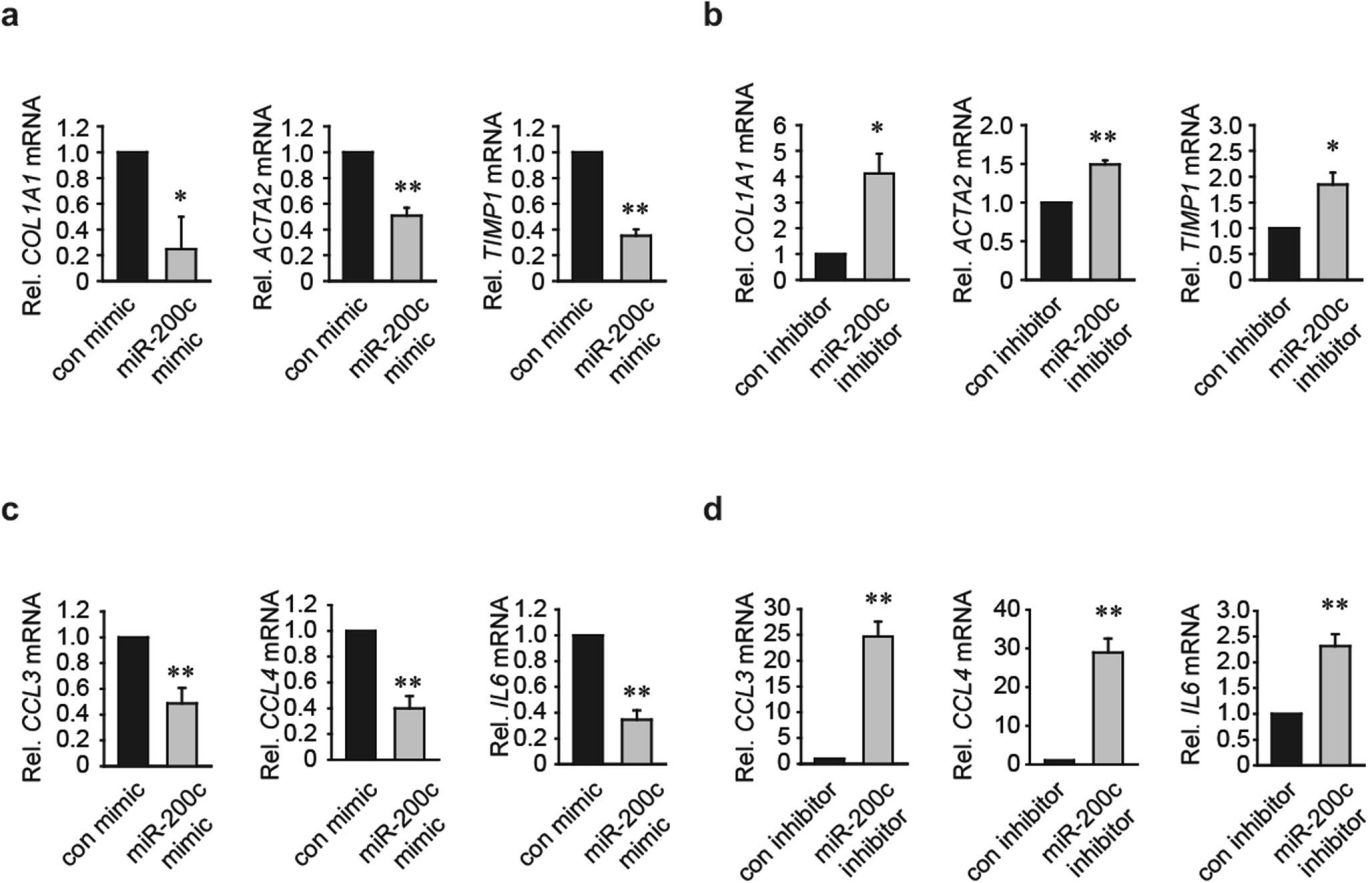
miR-200c decreases hepatic fibrogenic and inflammatory gene expression. **a** The effect of miR-200c mimic treatment on fibrogenic gene expression. LX-2 cells were transfected with the control mimic or miR-200c mimic. **b** qRT–PCR analysis of *COL1A1*, *ACTA2*, and *TIMP1* mRNA expression. LX-2 cells were transfected with the control inhibitor or miR-200c inhibitor. **c** Proinflammatory gene expression in LX-2 cells after miR-200c mimic transfection. **d** The effect of miR-200c inhibitor transfection on *CCL3*, *CCL4*, and *IL6* mRNA expression. *P < 0.05 and **P < 0.01 indicate a significant difference between the control (or inhibitor) and miR-200c mimic (or inhibitor) (*n* = 3–4 per group). The data are presented as the mean ± SEM values. Significance was assessed by two-tailed Student’s *t*-test.

To investigate the role of miR-200c in the fibrogenic response, we examined the effect of miR-200c on the expression of fibrogenic genes. After transfection with the miR-200c mimic, LX-2 cells expressed reduced levels of *COL1A1* (collagen, type I, alpha 1), *ACTA2* (encoding α-smooth muscle actin), and *TIMP1* (tissue inhibitor of metalloproteinases) (Fig. 3a). In contrast, the mRNA expression of these genes was upregulated by transfection with the miR-200c inhibitor (Fig. 3b). Next, we explored the role of miR-200c in chemokine and cytokine regulation. We found that miR-200c mimic transfection suppressed *CCL3, CCL4*, and *IL6* mRNA expression in LX-2 cells (Fig. 3c), whereas miR-200c inhibitor transfection had the opposite effect (Fig. 3d). These results indicate that miR-200c negatively regulates the expression of certain fibrogenic and inflammatory genes.

### Hepatic hyaluronidase-2 is overexpressed in advanced-stage liver fibrosis

**Fig. 4:**
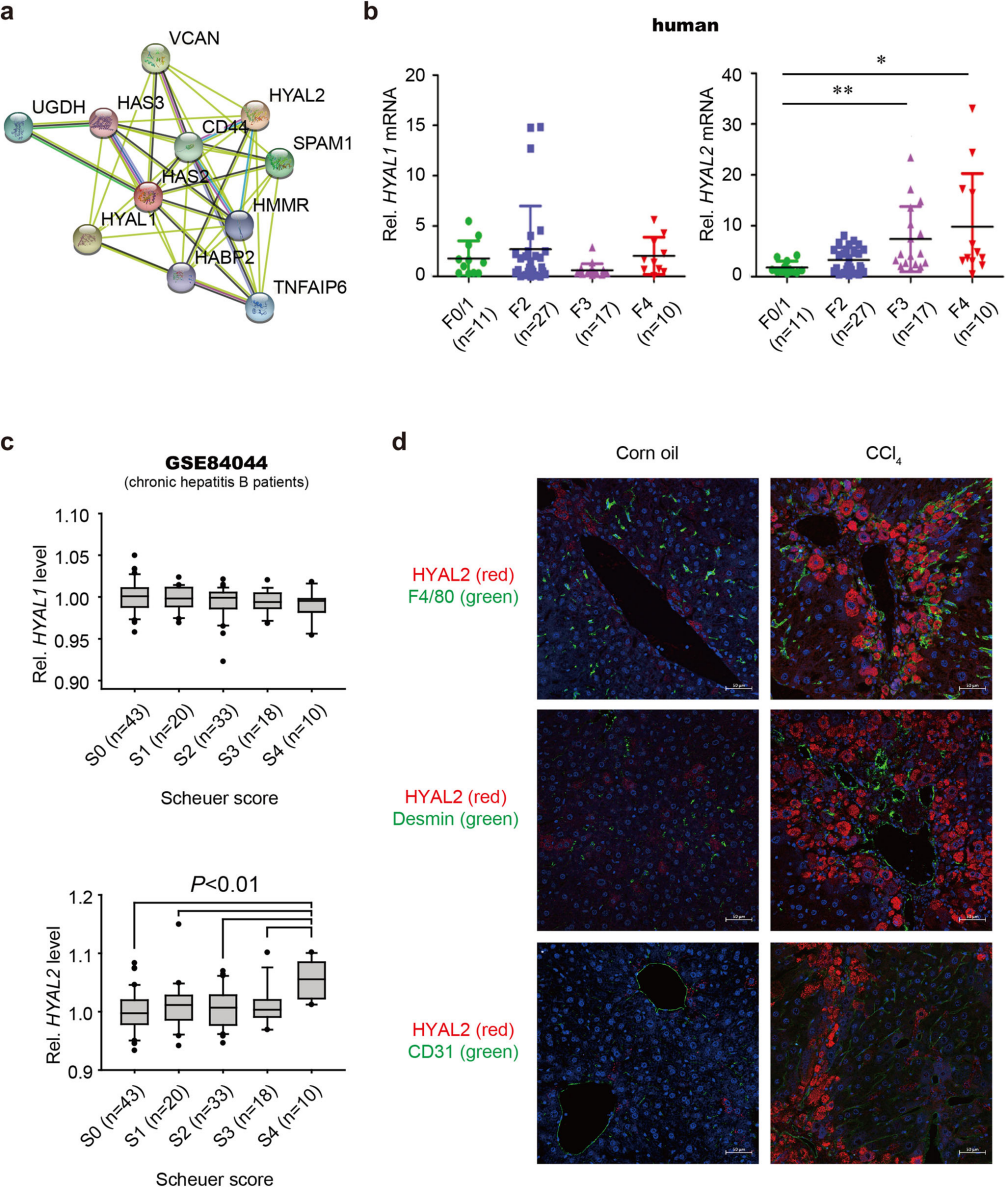
Hyaluronidase-2 expression is elevated in human liver fibrosis. **a** STRING protein–protein interaction network of HAS2. **b** *HYAL1* and *HYAL2* gene expression in liver samples from patients with fibrosis and chronic HBV. *P < 0.05 and **P < 0.01 indicate a significant difference versus F0/1. **c** Hepatic *HYAL1* and *HYAL2* expression in patients with HBV-related liver fibrosis (GEO accession number: GSE84044). Liver fibrosis was staged according to the Scheuer scoring system. The data are presented as the mean ± SEM values. Significance was assessed by one-way ANOVA with Tukey’s post-hoc test. **d** Mice were injected intraperitoneally with corn oil or CCl_4_ twice a week for 6 weeks. Representative immunofluorescence images of costaining for HYAL2 (red) and cell-specific markers (green)—F4/80 (a marker of macrophages), Desmin (a marker of HSCs), and CD31 (a marker of endothelial cells)—in the livers of corn oil- or CCl_4_-injected mice. Nuclei were stained with DAPI (blue). Scale bar, 50 μm.

LMW-HA is the predominant form of HA in the fibrotic liver and is the degradation product of HMW-HA produced by HAS2^15^. Using STRING, we predicted protein–protein interaction networks and found a functional association between HAS2 and the HA-degrading enzymes hyaluronidase 1 (HYAL1) and hyaluronidase-2 (HYAL2) (Fig. 4a). We assessed the expression of HYAL1 and HYAL2 in liver samples from our cohort of patients with chronic HBV infection and clinically diagnosed liver fibrosis. We observed increased hepatic expression of *HYAL2* but not *HYAL1* in advanced stages of fibrosis (F3 or F4) compared to early stages of fibrosis (F0/1) (Fig. 4b). Fibrosis can be staged on a scale from S0 to S4 according to the Scheuer scoring system. For additional validation, we analyzed another publicly available dataset (GSE84044) of HBV-induced fibrosis and found similar results (Fig. 4c). The basal mRNA expression of *Hyal1* and *Hyal2* in mouse liver cells is shown in Supplementary Fig. 1. Next, we performed double immunofluorescence staining for HYAL2 and cell-specific markers in the livers of mice with CCl_4_-induced liver fibrosis. In normal livers, HYAL2 expression was low, whereas in the livers of CCl_4_-treated mice, HYAL2 was overexpressed mainly in hepatocytes but not in F4/80^+^ Kupffer cells, Desmin^+^ HSCs, or CD31^+^ liver sinusoidal endothelial cells (Fig. 4d). Our results suggest that elevated HYAL2 expression may contribute to liver fibrosis.

### LMW-HA, but not HMW-HA, increases *Ccl3*, *Ccl4*, and *Tlr2* expression

**Fig. 5:**
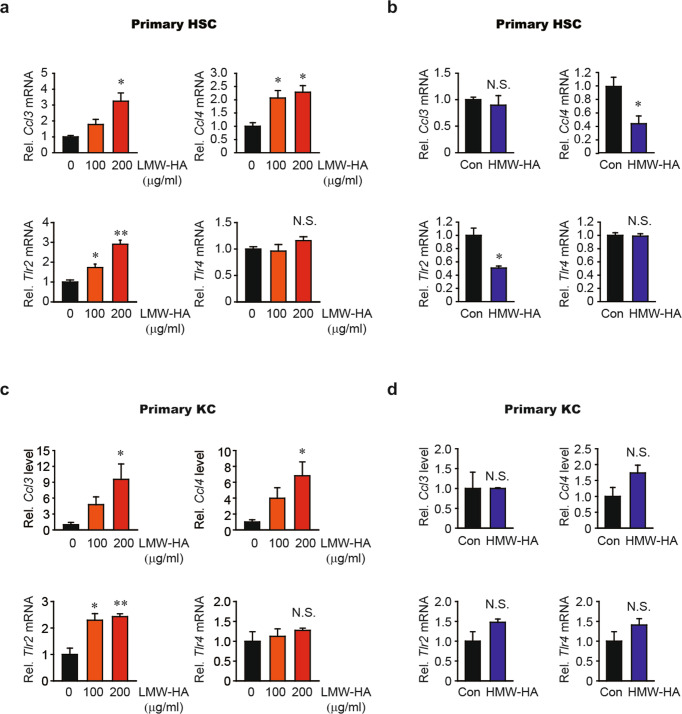
Low-molecular-weight HA elevates *Ccl3*, *Ccl4*, and *Tlr2* mRNA expression in HSCs and Kupffer cells. **a, b** *Ccl3*, *Ccl4*, *Tlr2*, and *Tlr4* mRNA expression in mouse hepatic stellate cells (HSCs). **a** Cells were treated with vehicle or with 100 or 200 μg/ml low-molecular-weight hyaluronan (LMW-HA) in combination with 10 μg/ml polymyxin B for 12 h. **b** Cells were treated with vehicle (Con) or 600 μg/ml high-molecular-weight hyaluronan (HMW-HA) in the presence of 10 μg/ml polymyxin B for 12 h. **c, d** qRT–PCR analysis of *Ccl3*, *Ccl4*, *Tlr2*, and *Tlr4* mRNA expression in mouse Kupffer cells (KC). Cells were treated with vehicle, with 100 or 200 μg/ml LMW-HA, or with 600 μg/ml HMW-HA in combination with 10 μg/ml polymyxin B for 4 h. *P < 0.05 and **P < 0.01 indicate a significant difference versus the Con group (*n* = 3 per group). N.S. not significant. The data are presented as the mean ± SEM values. Significance was assessed by one-way ANOVA with Tukey’s post-hoc test (**a, c**) and two-tailed Student’s *t*-test (**b, d**).

LMW-HA and HMW-HA have distinct roles in the regulation of chemokine gene expression^24^. LMW-HA often accumulates after tissue injury and plays a role in pathological processes such as inflammation and tissue remodeling^25^. In contrast, HMW-HA protects tissues from injury^26^. Because HYAL2 was overexpressed in fibrotic liver tissues and is expected to promote the accumulation of LMW-HA, we investigated the differential effects of LMW-HA and HMW-HA on chemokine production in primary mouse HSCs. LMW-HA treatment increased *Ccl3* and *Ccl4* mRNA expression in HSCs (Fig. 5a, upper). Moreover, LMA-HA enhanced the expression of the major HA receptor *Tlr2* but not that of *Tlr4* (Fig. 5a, lower). HMW-HA treatment had different effects on the expression of these mRNAs. HMW-HA reduced *Ccl4* and *Tlr2* mRNA expression in HSCs and did not affect *Ccl3* or *Tlr4* expression (Fig. 5b). We conducted identical experiments in primary mouse Kupffer cells and found similar effects. LMW-HA treatment significantly stimulated the expression of *Ccl3*, *Ccl4*, and *Tlr2* but not that of *Tlr4* (Fig. 5c). However, HMW-HA did not alter the expression of these genes (Fig. 5d). These results suggest that LMW-HA promotes an inflammatory response through the induction of chemokines, including CCL3 and CCL4.

### HSC-specific *Has2* deficiency attenuates acute CCl_4_-induced macrophage infiltration

**Fig. 6:**
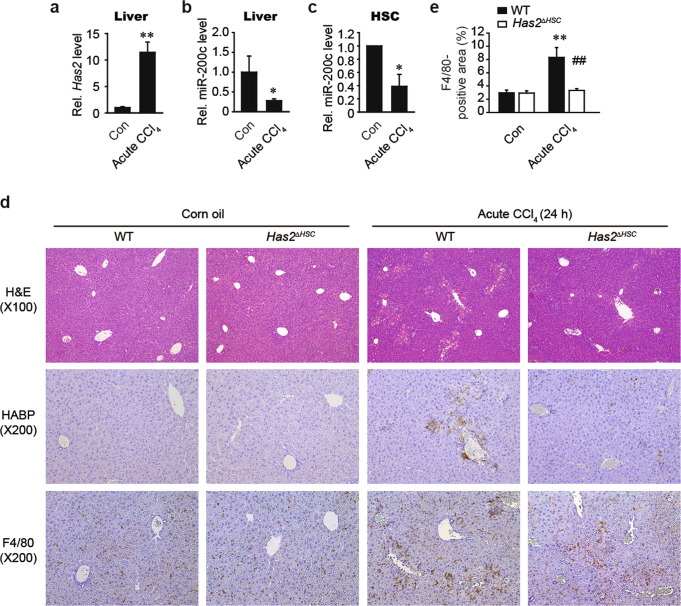
Has2 deficiency in hepatic stellate cells alleviates CCl4-induced ALI. C57BL/6 mice were injected intraperitoneally with CCl_4_ or corn oil (Con). Liver samples were harvested 24 h after CCl_4_ injection. **a** qRT–PCR analysis of *Has2* mRNA expression (Con, *n* = 5; CCl_4_, *n* = 7). **P < 0.01 indicates a significant difference versus the Con group. **b** qRT–PCR analysis of miR-200c expression in the livers of Con- and CCl_4_-treated mice. *P < 0.05 indicates a significant difference versus the Con group. **c** miR-200c levels in primary HSCs isolated from Con- and CCl_4_-treated mice. *P < 0.05 indicates a significant difference versus the Con group. **d** Representative images of H&E (upper), HA-binding protein (HABP) (middle), and F4/80 (lower) staining. **e** Quantification of F4/80^+^ staining. **P < 0.01 indicates a significant difference versus the WT-Con group; ^##^*P* < 0.01 indicates a significant difference versus the WT-CCl_4_ group. The data are presented as the mean ± SEM values. Significance was assessed by two-tailed Student’s *t*-test (**a–c**) and one-way ANOVA with Tukey’s post-hoc test (**e**).

Liver fibrosis results from repeated liver injury, which often involves hepatocyte death. Exposure to toxins, such as carbon tetrachloride (CCl_4_), also results in hepatocyte death and liver fibrosis. After injury or toxin exposure, HSCs migrate to and invade damaged liver tissue. At the site of tissue damage, HSCs engulf apoptotic bodies to alleviate liver injury^27^ while also stimulating profibrogenic responses^28^. We used a murine model of CCl_4_-induced liver injury to investigate the effect of HSC-derived HAS2 on liver pathology. A single injection of CCl_4_ increased the *Has2* mRNA level (Fig. 6a) and decreased the miR-200c level in the mouse liver (Fig. 6b). The level of miR-200c was also reduced in HSCs isolated from CCl_4_-treated mice (Fig. 6c). To determine the role of HAS2 in acute liver injury, HSC-specific *Has2* knockout (*Has2*^Δ*HSC*^) mice and control wild-type mice were administered a single dose of CCl_4_ and assessed for HA production and macrophage infiltration (F4/80 infiltration) (Fig. 6d). As expected, HSC-specific *Has2* deficiency decreased HA production (Fig. 6d, middle) and reduced macrophage infiltration (Fig. 6d, e). Taken together, our results suggest that *Has2* deficiency in HSCs ameliorates acute CCl_4_-induced liver inflammation.

### HSC-derived *Has2* plays a key role in CCl_4_-induced chronic inflammation and fibrosis

**Fig. 7:**
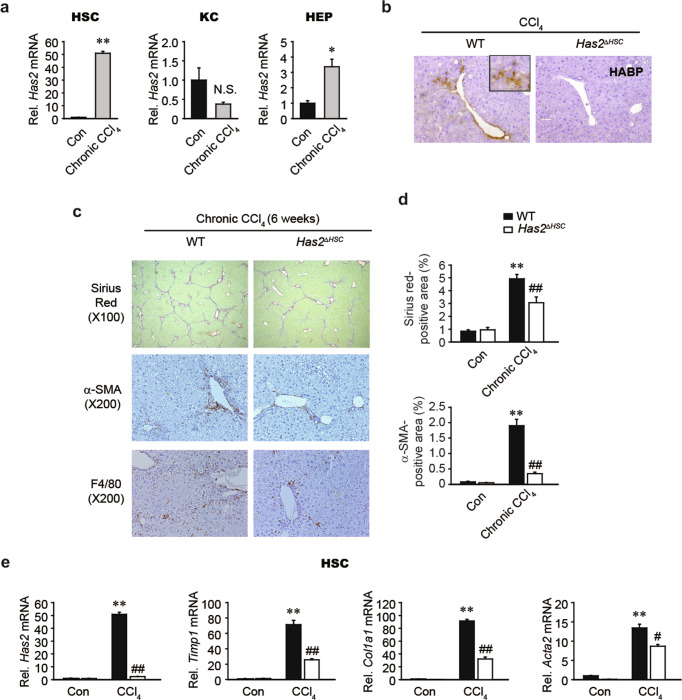
Has2 deficiency in hepatic stellate cells attenuates CCl4-induced chronic liver fibrosis and inflammation. **a** qRT–PCR analysis of *Has2* mRNA expression in primary hepatic stellate cells (HSC), Kupffer cells (KC), and hepatocytes (HEP) isolated from corn oil (Con)- and CCl_4_-treated mice. Mice were injected intraperitoneally with corn oil or CCl_4_ twice a week for 3 weeks. Cells were isolated two days after the last injection. *P < 0.05 and **P < 0.01 indicate a significant difference versus the Con group. N.S. not significant. Significance was assessed by two-tailed Student’s *t-*test. **b** Representative images of HA staining of mouse liver sections. Wild-type (WT) and HSC-specific *Has2* knockout (*Has2*^Δ*HSC*^) mice were treated with CCl_4_ twice a week for 6 weeks (*n* = 5–6 mice per group). **c** Sirius Red staining and immunohistochemical staining for α-SMA and F4/80. Representative images are shown. **d** Quantification of the Sirius Red^+^ area and α-SMA^+^ area. **e** qRT–PCR analysis of *Has2*, *Timp1*, *Col1a1*, and *Acta2* mRNA expression in primary HSCs (*n* = 3 per group). **P < 0.01 indicates a significant difference versus the WT-Con group; ^#^*P* < 0.05 and ^##^*P* < 0.01 indicate a significant difference versus the WT-CCl_4_ group. The data are presented as the mean ± SEM values. Significance was assessed by one-way ANOVA with Tukey’s post-hoc test.

To extend our observations from acute liver injury to chronic liver fibrosis, we subjected mice to repeated CCl_4_ treatment. In wild-type mice, chronic CCl_4_ treatment dramatically increased *Has2* mRNA expression in HSCs and hepatocytes but not in Kupffer cells (Fig. 7a). *Has2* mRNA expression showed a four-fold increase in hepatocytes from chronic CCl_4_-treated mice compared to control mice, which was a lesser degree of upregulation than observed in HSCs (Fig. 7a). To induce liver fibrosis, CCl_4_ was administered twice a week for 6 weeks. HSC-specific *Has2* deletion dramatically reduced HA accumulation in the livers of mice after repeated injections of CCl_4_ (Fig. 7b). We then examined the role of HSC-derived *Has2* in CCl_4_-induced liver fibrosis. *Has2*^Δ*HSC*^ mice showed a reduced degree of collagen deposition (a hallmark of liver fibrosis) and reduced HSC activation, as shown by Sirius Red staining and α-SMA staining, respectively (Fig. 7c, d). We also examined the accumulation of liver macrophages, which contribute to tissue repair, liver inflammation, and fibrosis, by staining tissues for F4/80^29^. Immunohistochemical staining for F4/80 showed that macrophage infiltration was lower in the livers of CCl_4_-treated *Has2*^Δ*HSC*^ mice than in those of CCl_4_-treated wild-type mice (Fig. 7d). Finally, *Has2* deficiency in HSCs downregulated CCl_4_-induced *Timp1*, *Col1a1*, and *Acta2* mRNA expression (Fig. 7e). These findings indicate that HSC-derived *Has2* contributes to CCl_4_-induced liver inflammation and fibrosis.

### Genetic deletion of *Has2* in HSCs and pharmacological inhibition of hyaluronan synthesis by 4-MU decreases BDL-induced *Ccl3* and *Ccl4* expression

**Fig. 8:**
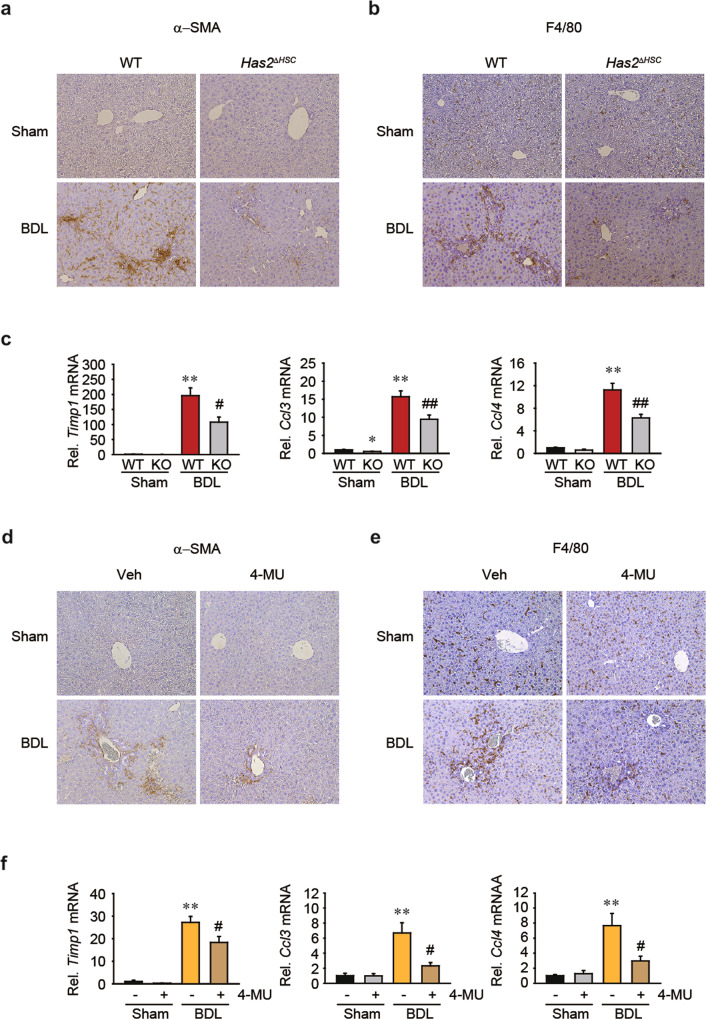
Has2 deficiency in hepatic stellate cells or treatment with 4-methylumebelliferone decreases *Ccl3* and *Ccl4* mRNA levels after bile duct ligation. **a** Immunohistochemical staining for α-SMA in liver sections of wild-type (WT) and *Has2*^Δ*HSC*^ mice 3 weeks after bile duct ligation (BDL). **b** Immunohistochemical staining for F4/80. Representative images are shown. **c** qRT–PCR analysis of *Timp1*, *Ccl3*, and *Ccl4* in the livers of mice. *P < 0.05 and **P < 0.01 indicate a significant difference versus the Sham-WT group; ^#^*P* < 0.05 and ^##^*P* < 0.01 indicate a significant difference versus the BDL-WT group (*n* = 3–11 mice per group). The data are presented as the mean ± SEM values. Significance was assessed by one-way ANOVA with Tukey’s post-hoc test. **d** Immunohistochemical staining for α-SMA in mouse livers 5 days after BDL. Sham- and BDL-operated mice received 225 mg/kg 4-methylumbelliferone (4-MU) or vehicle (Veh) twice a day for 5 days orally. **e** Immunohistochemical staining for F4/80. Representative pictures are shown. **f** Hepatic *Timp1*, *Ccl3*, and *Ccl4* mRNA levels were determined by quantitative PCR. **P < 0.01 indicates a significant difference versus the Sham-Veh group; ^#^*P* < 0.05 indicates a significant difference versus the BDL-Veh group (*n* = 4–9 mice per group). The data are presented as the mean ± SEM values. Significance was assessed by one-way ANOVA with Tukey’s post-hoc test.

Previously, we showed that BDL-induced liver fibrosis is ameliorated in *Has2*^Δ*HSC*^ mice compared to wild-type mice^15^. BDL-induced liver fibrosis develops as a consequence of cholestatic liver injury. We used tissues harvested from mice in our previous study to evaluate α-SMA and F4/80 expression. After BDL, the populations of α-SMA^+^ cells (activated HSCs) and F4/80^+^ cells (macrophages) were reduced in *Has2*^Δ*HSC*^ mice compared to wild-type mice (Fig. 8a, b). HSC-specific deficiency of *Has2* significantly inhibited the BDL-induced upregulation of *Timp1, Ccl3*, and *Ccl4* mRNA expression (Fig. 8c).

To investigate the effect of HA synthesis on liver fibrosis, we treated mice with the HA synthesis inhibitor 4-MU after BDL. Treatment with 4-MU also reduced the populations of α-SMA^+^ cells (activated HSCs) and F4/80^+^ cells (macrophages) in the livers of BDL mice (Fig. 8d, e). Moreover, 4-MU treatment decreased BDL-induced *Timp1*, *Ccl3*, and *Ccl4* mRNA expression (Fig. 8f). Together, these results demonstrate that inhibition of HSC-derived HAS2 activity and HA production may suppress liver inflammation and fibrosis.

## Discussion

The GBD 2017 Cirrhosis Collaborators reported that the number of deaths and disability-adjusted life-years due to liver cirrhosis increased (146%) from 1990 to 2017^1^. Thus, cirrhosis is a growing global health burden. Cirrhosis is an advanced form of liver fibrosis, which can be initiated by a variety of factors, including hepatitis virus infection, excessive alcohol consumption, metabolic syndrome, and environmental toxin exposure. These insults induce a fibrotic response characterized by excessive extracellular matrix production and accumulation^30^. During liver fibrosis, HAS2 expression is substantially induced in HSCs^15^. Here, we found that HAS2 was upregulated in a murine model of CCl_4_-induced acute liver injury (Fig. 6a). Due to its association with extracellular matrix-producing HSCs and liver fibrosis, HAS2 is an attractive target for antifibrotic therapies. To develop such therapies, in-depth knowledge of the regulation of HAS2 is necessary.

HAS2 transcription is regulated by the transcription factor TBX4 and promotes myofibroblast accumulation and pulmonary fibrosis^31^. Sonic hedgehog signaling also regulates HAS2 transcriptionally in the limb bud^32^, and gli-regulated HAS2 expression contributes to the positioning of joint progenitor cells^32^. In liver fibrosis, WT1 binds to the HAS2 promoter and stimulates the transcription of HAS2^15^. In contrast to these well-defined transcriptional networks, the posttranscriptional regulation of HAS2 in liver fibrosis has not been elucidated. Here, we demonstrated that downregulation of miR-200c contributes to the progression of liver fibrosis by regulating HAS2 expression posttranscriptionally.

The expression of miRNAs is often dysregulated in disease. Because a single miRNA can target multiple genes, dysregulated miRNAs can have a broad impact on pathophysiology. Numerous studies have shown that miRNAs contribute to a spectrum of liver diseases, including liver injury, inflammation, fibrosis, and tumorigenesis^33^. Therefore, miRNAs are attractive as biomarkers and therapeutic targets for liver diseases^34,35^. Here, we investigated the posttranscriptional regulation of HAS2 by miRNAs. MiRNAs are 18–25 nucleotides in length and contain 6–8 nucleotides at the 5’ end. These regions are complementary to the seed sequence in their target mRNAs^36^. TargetScan is a representative miRNA target prediction algorithm that is used to search for seed matches. We used TargetScan to identify miRNAs that may interact with the 3’UTR of HAS2. Among the candidate miRNAs, miR-200c was the only miRNA downregulated in liver fibrosis in both humans and mice (Fig. 1). MiR-200c is a member of the miR-200 family (i.e., miR-141, miR-200a, miR-200b, miR-200c, and miR-429) and primarily functions as a tumor suppressor. Altered expression of miR-200c in hepatocellular carcinoma is associated with poor prognosis^12^. Mechanistically, TGF-β treatment induces DNA methylation of the miR-200 family promoters, effectively silencing the expression of these miRNAs^37^. TGF-β suppresses the miR-200 cluster, and suppression of miR-200c enhances TGF-β production. The relationship between miR-200c and TGF-β is important because TGF-β promotes EMT processes through an autocrine TGF-β/ZEB/miR-200 regulatory loop^37^. Moreover, TGF-β is a key fibrotic regulator in liver fibrosis and stimulates the expression of *Col1a1*, *Acta2*, and *Timp1*. In our study, inhibition of miR-200c upregulated these fibrogenic genes (Fig. 3b).

The expression of miR-200c is frequently downregulated in liver diseases, and low levels of miR-200c are associated with poor overall survival in hepatocellular carcinoma patients^38^. Moreover, miR-200c inhibits HBV replication and gene expression by targeting nuclear factor 1A^39^. In turn, HBV suppresses miR-200c transcription by reactivating the oncofetal protein Sal-like protein 4 (SALL4), a zinc-finger transcription factor^38^. As PD-L1 is a direct target of miR-200c, downregulation of miR-200c by HBV results in increased expression of PD-L1 and CD8^+^ T-cell exhaustion^38^. miR-200c expression is also reduced in patients with nonalcoholic fatty liver disease^40^. Reduced miR-200c levels are associated with hepatic lipid accumulation via targeting of Jun, which is a transcription factor of SREBP1, a key regulator of de novo lipogenesis^40^. Here, we found that miR-200c expression was downregulated in both CCl_4_- and BDL-induced liver fibrosis (Fig. 1). CCl_4_ is one of the most commonly used liver toxins to induce liver injury and fibrosis. In hepatocytes, CCl_4_ is metabolized into a toxic trichloromethyl (CCl_3_^−^) radical by CYP2E1, which causes lipid peroxidation and, ultimately, centrilobular necrosis^41^. Repeated injection of CCl_4_ results in liver fibrosis initiated from pericentral regions, followed by bridging fibrosis. The BDL model is a representative biliary fibrosis model. BDL promotes the proliferation of biliary epithelial cells and oval cells, leading to ductular reaction, cholestasis, portal inflammation, and fibrosis^41^. Due to the different pathogeneses of liver fibrosis, the molecular profile can be different between the CCl_4_ and BDL models. The Kisseleva group investigated the gene expression profile of CCl_4_-activated HSCs, BDL-activated HSCs, and BDL-activated portal fibroblasts^42^. These three different cell types had overlapping gene signatures but also distinct gene expression profiles. We also found that miR-200b, miR-29a, and miR-190a/b were differentially regulated in the CCl_4_ and BDL models (Fig. 1d, e).

More importantly, miR-200c was suppressed in both in vivo fibrosis models. We also analyzed the miRNA profiles of clinical liver fibrosis samples from the GSE49012 dataset. Consistent with our murine models of liver fibrosis, miR-200c expression was reduced in the fibrotic samples in the human dataset (GSE49012). Together with those of the present study, these findings suggest that deregulation of miR-200c could be an early event in liver fibrosis. Indeed, we found that a single injection of CCl_4_ significantly decreased miR-200c expression (Fig. 6). A recent study demonstrated that loss of miR-200c accelerated intrahepatic inflammation and periductular fibrosis by targeting SESN1 and repressing the IL-6/AKT loop in cholestatic liver fibrosis models^43^. Our in vitro experiments also indicated that miR-200c downregulated fibrogenic and proinflammatory gene expression (Fig. 3). Collectively, these findings indicate that miR-200c can reduce inflammation and fibrosis via the regulation of IL-6 and HAS2.

Moreover, the expression of miR-200c and HAS2 was reciprocally regulated in both acute liver injury and chronic liver fibrosis. Mechanistically, we showed that HAS2 is a novel target of miR-200c. HAS enzymes (HAS1, HAS2, and HAS3) generate HMW-HA (up to 1000 kDa). Of the three HAS enzymes, only HAS2 can synthesize HA chains as large as 6000 kDa^44^. Under exposure to fibrotic stimuli, HA is largely produced by HAS2 in HSCs^15^. Enzymatically, HMW-HA is cleaved by hyaluronidases into LMW-HA, which is the predominant form of HA in liver fibrosis^15^. HYAL2 but not HYAL1 was upregulated in patients with advanced-stage liver fibrosis (Fig. 4b, c). Similarly, the expression of HYAL2 was higher in the fibrotic livers of CCl_4_-treated mice (Fig. 4d). Together, these findings suggest that HYAL2 contributes to the abundance of LMW-HA in liver fibrosis.

LMW-HA regulates proinflammatory chemokines such as CCL2, CCL3, and CCL4^24,25^, which we further confirmed in our models (Fig. 5). CCR2 (a receptor for CCL2) and CCR5 (a receptor for CCL3 and CCL4) have been proposed to be targets for antifibrotic therapy due to their ability to promote liver fibrosis and macrophage infiltration^20,45^. For example, treatment with a CC chemokine neutralizer was found to prevent the development of liver fibrosis in mice^20^. Additionally, administration of a CCR2 inhibitor was found to prevent macrophage infiltration, thereby inhibiting liver inflammation and fibrosis in nonalcoholic steatohepatitis^46^. Cenicriviroc, a CCR2/CCR5 inhibitor, is being used in clinical trials for the treatment of liver fibrosis in nonalcoholic steatohepatitis patients^47^. Here, we explored the therapeutic potential of HAS2 inhibition in acute liver injury and liver fibrosis. Genetic ablation of *Has2* in HSCs prevented CCl_4_-induced acute liver injury and inflammation (Fig. 6). HSC-specific loss of *Has2* also ameliorated chronic CCl_4_-induced hepatic inflammation and fibrosis (Fig. 7). We investigated the role of HA in a BDL model, another representative liver fibrosis model. Genetic ablation of *Has2* in HSCs inhibited BDL-induced activation of HSCs and expression of *Ccl3* and *Ccl4* (Fig. 8). Macrophage infiltration was also reduced in BDL-operated *Has2*^Δ*HSC*^ mice compared with BDL-operated wild-type mice (Fig. 8). Finally, pharmacological inhibition of HA synthesis with 4-MU showed similar results. Our in vivo animal studies demonstrated that the expression of HAS2 contributes to acute liver injury, liver inflammation, and fibrosis, suggesting that targeting HAS2 could improve liver pathology.

In conclusion, we demonstrated that miR-200c expression is downregulated in acute and chronic liver injury and directly targets HAS2. We also note that HAS2 and HYAL2 play important roles in the production and degradation of HA in the fibrotic liver. Collectively, our findings suggest that downregulation of miR-200c in the fibrotic liver allows HAS2 to synthesize excess HA, which induces the secretion of chemokines, ultimately attracting macrophages to the injured liver and perpetuating an inflammatory microenvironment (Supplementary Fig. 2). The present study provides novel insight into the molecular regulation of HAS2 and may identify a novel therapeutic target for liver inflammation and fibrosis.

## Supplementary information

Supplementary Table 1 and Figure 1&2
