# Supplementary material for: Hyaluronan synthase 2, a target of miR-200c, promotes carbon tetrachloride-induced acute and chronic liver inflammation via regulation of CCL3 and CCL4
Source: Exp Mol Med. 2022 Jun 3;54(6):739–52. doi: 10.1038/s12276-022-00781-5 (PMC9256637; doi:10.1038/s12276-022-00781-5)
Supplement: Supplementary file 1 — Supplementary Table 1 and Figure 1&2 [file 12276_2022_781_MOESM1_ESM.pdf]

Supplementary Table 1. List of primer sequences used for RT-PCR analysis

Gene	Forward	Reverse
18S rRNA	AGTCCCTGCCCTTTGTACACA	CGATCCGAGGGCCTCACTA
mActa2	ACTGGGACGACATGGAAAAG	GTTCAGTGGTGCCTCTGTCA
mCcl3	ACCATGACACTCTGCAACCA	GTGGAATCTTCCGGCTGTAG
mCcl4	CATGAAGCTCTGCGTGTCTG	GAAACAGCAGGAAGTGGGAG
mCol1a1	ACATGTTCAGCTTTGTGGACC	TAGGCCATTGTGTATGCAGC
mHas2	TGAGCAGGAGCTGAACAAGA	GCCAACAATATAAGCAGCTGTG
mHyal1	AAGTACCAAGGAATCATGCC	CTCAGGATAACTTGGATGGC
mHyal2	GGTGGACCTTATCTCTACCAT	TATTGGCAGGTCTCCATACTT
mTimp1	GTAAGGCCTGTAGCTGTGCC	AGGTGGTCTCGTTGATTTCT
mTlr2	CTCCCACTTCAGGCTCTTTG	AGGAACTGGGTGGAGAACCT
mTlr4	TGTTCTTCTCCTGCCTGACA	TGTCATCAGGGACTTTGCTG
hACTA2	CCAAGCACTGTCAGGAAT	AGGCAGTGCTGTCCTCTT
hCCL3	TCAGACTTCAGAAGGACACGG	CTGCATGATTCTGAGCAGGTG
hCCL4	GCAAGTCTGTGCTGATCCCA	GCGGAGAGGAGTCCTGAGTA
hCOL1A1	AACATGACCAAAAACCAAAAGTG	CATTGTTTCCTGTGTCTTCTGG
hHAS2	ACAGACAGGCTGAGGACGAC	AGCTGTGATTCCAAGGAGGA
hHYAL1	TGGTCACGTTCAGGATGAAG	GTGCTGCCCTATGTCCAGAT
hHYAL2	GTCACCCCAGAGGATGACAC	TACGTCTTCACACGACCCAC
hTIMP1	CTTCTGCAATTCCGACCTCGT	ACGCTGGTATAAGGTGGTCTG
miR-29a	UAGCACCAUCUGAAAUCGGUUA	
miR-190a	TGATATGTTTGATATATTAGGT	
miR-190b	TGATATGTTTGATATTGGGTTG	
miR-200b	TAATACTGCCTGGTAATGATGA	
miR-200c	TAATACTGCCGGGTAATGATGGA	
U6	GGGCAGGAAGAGGGCCTAT	

Abbreviation: RT-PCR, reverse transcription-polymerase chain reaction

Supplementary Figures

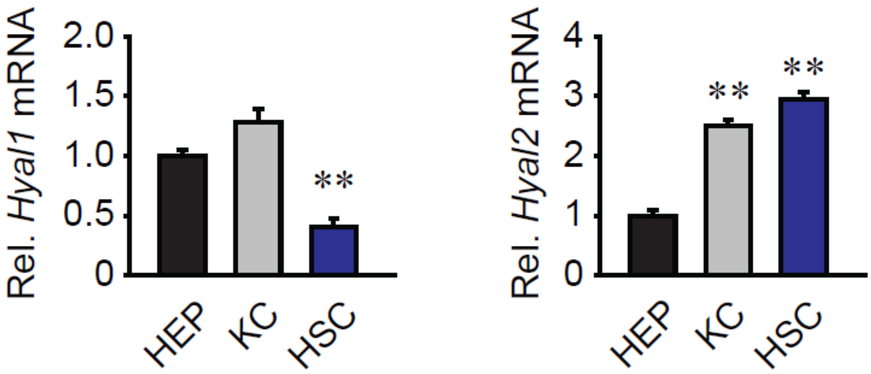

Supplementary Fig. 1 qRT-PCR assays for *Hyal1* and *Hyal2* mRNA in primary mouse hepatocytes (HEP), Kupffer cells (KC), and hepatic stellate cells (HSC).

***P*<0.01 significantly different from HEP. Data are presented as the mean±SEM. Significance was assessed by one-way ANOVA with Tukey's post hoc analysis. (*n* = 3)

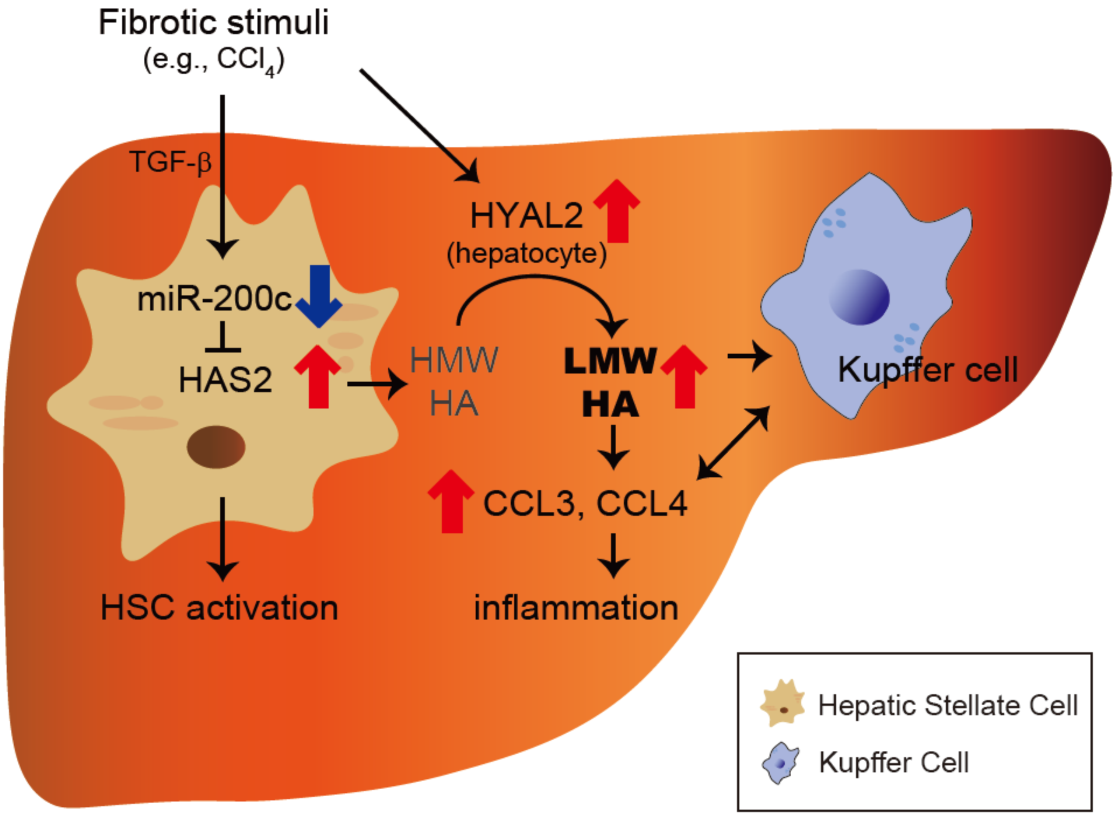

Supplementary Fig. 2 Schematic illustration of the proposed model for miR-200c-HAS2-CCL3 and CCL4 in acute and chronic liver inflammation.

MiR-200c is decreased by fibrotic stimuli, which is responsible for the increase of HAS2 in hepatic stellate cells (HSCs). Fibrotic stimuli increase Hyaluronidase 2 (HYAL2) in hepatocytes, resulting in the elevation of low molecular weight hyaluronan (LMW-HA). LMW-HA stimulates the gene expression of *Ccl3* and *Ccl4* in HSCs and Kupffer cells, ultimately recruiting macrophages to the injured liver and promoting liver inflammation.
